# Statins Alleviate Tumor Hypoxia in Prostate Cancer Models by Decreasing Oxygen Consumption: An Opportunity for Radiosensitization?

**DOI:** 10.3390/biom12101418

**Published:** 2022-10-03

**Authors:** Donatienne d’Hose, Lionel Mignion, Loïc Hamelin, Pierre Sonveaux, Bénédicte F. Jordan, Bernard Gallez

**Affiliations:** 1 Biomedical Magnetic Resonance, Louvain Drug Research Institute (LDRI), Université Catholique de Louvain (UCLouvain), 1200 Brussels, Belgium; 2 Pole of Pharmacology and Therapeutics, Institut de Recherches Expérimentales et Cliniques (IREC), Université Catholique de Louvain (UCLouvain), 1200 Brussels, Belgium; 3 Walloon Excellence in Life Sciences and Biotechnology (WELBIO) Research Institute, 1300 Wavre, Belgium

**Keywords:** statins, tumor hypoxia, oxygen, EPR, irradiation, oxygen consumption, superoxide, cancer metabolism

## Abstract

Background: Because statins were found to decrease the oxygen consumption rate (OCR) of a variety of normal cells, our hypothesis was that statins may also decrease the OCR of cancer cells, alleviate tumor hypoxia and radiosensitize tumors. Methods: OCR was assessed using the Seahorse XF96 technology and EPR respirometry in PC-3 prostate cancer cells. Mitochondrial superoxide production was measured by EPR with mitoTEMPO-H as a sensing probe. Tumor pO_2_ was measured in vivo using low-frequency EPR oximetry to define the optimal window of reoxygenation, the time at which tumors were irradiated with a single 6 Gy dose with a Cesium-137 irradiator. Results: 24-h exposure to simvastatin and fluvastatin significantly decreased the OCR of PC-3 cancer cells. An increase in mitochondrial superoxide levels was also observed after fluvastatin exposure. The PC-3 prostate cancer model was found highly hypoxic at the basal level. When mice were treated with simvastatin or fluvastatin (daily injection of 20 mg/kg), tumor oxygenation increased 48 and 72 h after initiation of the treatment. However, despite reoxygenation, simvastatin did not sensitize the PC-3 tumor model to RT. Conclusions: exposure to statins affect tumor metabolism and tumor oxygenation, however, with limited impact on tumor growth with or without irradiation.

## 1. Introduction

Statins are cholesterol-lowering drugs widely used in the prevention of cardiovascular diseases. Statins inhibit HMG-CoA reductase, and thus decrease the biosynthesis of low-density lipoprotein cholesterol. Evidence from experimental and preclinical studies [1,2] has indicated that statins can inhibit tumor growth and induce apoptosis in several tumor types, including pancreatic carcinoma [3], breast cancer [4], small-cell lung cancer [5] and brain tumors [6]. Mechanistically, it has been suggested that, because statins reduce mevalonate synthesis, farnesylation and geranylgeranylation are decreased. As a result, there is a decrease in Ras, Rho, and c-Myc protein expression, which reduces tumor cell proliferation and migration. Statins also reduce the expression of matrix metalloproteinase-9 involved in metastasis, inhibit angiogenesis by increasing the inhibitory effect of TNF alpha, and activate caspase-9 and caspase-3, which leads to apoptosis [2]. The potential benefit of statins in cancer prevention and treatment has recently received significant interest with the aim to evaluate the impact of statins on the outcome of cancer treatments. In an analysis of about one thousand patients with a diagnosis of colon cancer, authors associated statin administration with a reduced risk of death from any cause or from cancer [7]. Another meta-analysis suggested that the use of statins is associated with a reduced mortality and recurrence of hepatocellular carcinomas [8], and improved survival for rectal cancer [9], head and neck cancer [10], and lung cancer [11] patients. However, other studies reported no clinical benefit from the use of statins in esophageal cancer [12], colorectal cancer [13], or small-cell lung cancer [14]. Interestingly, the statin studies that failed were either population based or a randomized phase III trial, whereas those that were positive were based on meta-analyses.

Interestingly, several large-scale cohort studies have recently indicated a better outcome of radiation therapy (RT) (external or brachytherapy) in prostate cancer patients under statin treatment [15,16,17]. A meta-analysis evaluated associations between statins and recurrence-free survival following the treatment of localized prostate cancer, and found a beneficial effect of statins for prostate cancer patients treated with RT but not for radical prostatectomy patients [18]. These clinical observations warrant further research on the potential mechanisms underlying a potential radiosensitizing property of statins. Interestingly, it was suggested that atorvastatin could enhance radiosensitivity in hypoxia-induced prostate cancer cells, an effect that could be due to the decrease in the expression of HIF-1α in hypoxic cells [19].

Our hypothesis is that the alleviation of tumor hypoxia induced by an inhibition of the oxygen consumption rate (OCR) of cancer cells could play a role in the improved response to RT in those patients receiving statins. It is important to remember that most malignant solid tumors contain areas with hypoxia resulting in worsened clinical prognosis for cancer patients [20,21,22,23,24]. Tumor hypoxia results from an imbalance between the limited oxygen delivery capacity of the abnormal vasculature and the high OCR of tumor cells [23]. Tumor hypoxia is considered to be a crucial therapeutic issue, as it renders solid tumors resistant to radiation therapy. Because of the so-called “Oxygen Enhancement Effect”, the radiation dose required to achieve the same biological effect is about three times higher in hypoxic tissues (less than 10 mm Hg) than in those with normal oxygen levels [25]. Tumor hypoxia may theoretically be alleviated by increasing either oxygen delivery or by decreasing the OCR of cancer cells. Mathematical modelling described by Secomb et al. suggested that tumor hypoxia can be abolished by an OCR reduction of about 30% [26]. Experimentally, several strategies designed to decrease the OCR of cancer cells succeeded in increasing the response to radiation therapy in tumor models [27,28,29,30,31,32,33]. The effect of statins on OCR has been demonstrated in a variety of normal cells, such as cardiomyocytes, skeletal muscle cells, and hepatocytes [34,35,36]. It is generally admitted that this effect is mediated by a reduction of the synthesis of ubiquinol (Coenzyme Q10). So far, only one study investigated the effect of statins on the OCR of cancer cells. This study evaluated the impact of lovastatin (in comparison with fludarabin) on DS sarcoma cells with the aim to evaluate the impact on DNA synthesis and OCR [37]. However, these authors did not investigate the consequences of the changes in OCR on the sensitivity to irradiation. 

**Figure 1:**
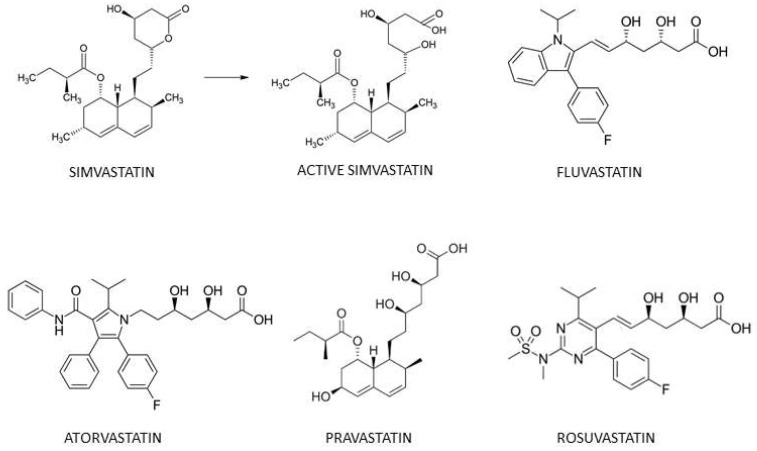
Structure of statins used in the present study: simvastatin, the active metabolite of simvastatin, fluvastatin, atorvastatin, rosuvastatin and pravastatin.

The overall aim of the present study was to evaluate the impact of statins on cancer cell OCR, tumor hypoxia and the response to irradiation. For this purpose, we first screened the effect of five statins (simvastatin, fluvastatin, rosuvastatin, pravastatin and atorvastatin; Figure 1) on the OCR of prostate cancer cells. Using the Seahorse XF96 technology, we observed that a 24-h exposure of prostate cancer cells to simvastatin and fluvastatin significantly decreased their basal OCR, while the other statins did not decrease OCR. These results were confirmed using Electron Paramagnetic Resonance (EPR)-based OCR measurements. Because alterations of the electron transport chain may lead to an increase in superoxide production, we further investigated the impact of statins on the production of mitochondrial superoxide using EPR spectrometry. In addition, we performed quantitative measurements of tumor oxygenation in the PC-3 prostate cancer model in mice. When treated by simvastatin or fluvastatin (daily injection of 20 mg/kg), tumor oxygenation significantly increased 48 and 72 h after starting the treatment. Irradiation experiments were carried out to analyze the potential radiosensitizing properties of statins at the time of maximal reoxygenation.

## 2. Materials and Methods

### 2.1. Reagents

Simvastatin (CAS 79902-63-9) was from Enzo Lifescience (Antwerpen, Belgium), whereas Atorvastatin (CAS 344423-98-9, catalog #PHR1422), Pravastatin (CAS 81131-70-6), Rosuvastatin (CAS 147098-20-2), and Fluvastatin (CAS 93957-55-2) were purchased from Sigma Aldrich (Overijse, Belgium). EPR probes ^15^N-PDT (4-oxo-2,2,6,6-tetramethylpiperidine-d_16_-^15^N-1-oxyl) and MitoTEMPO-H (1-hydroxy-4-[2-triphenylphosphonio)-acetamido]-2,2,6,6-tetramethylpiperidine) originated from CDN Isotopes and Enzo Lifescience, respectively. Dimethyl sulfoxide (DMSO), superoxide dismutase conjugated with polyethylene glycol (PEGSOD2), diethylenetriaminepentaacetic acid (DTPA) and dextran from leuconostoc mesenteroides (average MW 60,000–76,000) were from Sigma-Aldrich (Hoeilaert, Belgium).

### 2.2. Cell Line and Culture

The PC-3 cell line was purchased from the American Type Culture Collection (Manassas, VA, USA), and maintained in cell culture incubators (humidified atmosphere with 5% CO_2_ at 37 °C). They were routinely cultured in Ham’s F-12K (Kaighn’s) medium with 10% heat-inactivated fetal bovine serum (FBS; Thermo Fisher Scientific, Merelbeke, Belgium). 

### 2.3. OCR Measurements by the Seahorse XF96 Technology

OCR measurements were performed using a XF Cell Mitostress test kit (Agilent, Santa Clara, CA, USA) on a Seahorse XF96 bioenergetic analyzer (Agilent) [38] according to manufacturer’s instructions. Briefly, cells were first seeded at 10,000 cells/well into a Seahorse XF96 V3 PS plate 24 h before treatment. They were then treated for 2 or 24 h with statins or control solution (DMSO 0.1%). Before the assay, cell medium was replaced by the conditional medium without sodium bicarbonate (Dilution of Dulbecco’s modified Eagle’s medium (Cat. D5030, Sigma-Aldrich) supplemented with 1.85 g/L NaCl, phenol red, 10 mM D-glucose, 2 mM L-glutamine, 2 mM Pyruvate and 0.5% FBS) with or without statin, and incubated at 37 °C without CO_2_ for 1 h before completion of probe cartridge calibration. The following drugs were successively injected: oligomycin (1 M), FCCP (2 M) and Rotenone/Antimycin A (0.5 M). Data were visualized and analyzed using Agilent Wave Software. Results were normalized to the total number of cells determined using a SpectraMax i3 plate imager (Molecular Devices, Sunnyvale, CA, USA) and SoftMax Pro software (Molecular Devices). Basal mitochondrial OCR was determined by subtracting the OCR measured after injection of Rotenone/Antimycin A (=non-mitochondrial respiration) to OCR measured before any drug injection. Concentrations of statins used in the experiments were inspired by publications searching for mechanisms that could explain the potentiation of anti-cancer activity induced by statins (10 µM) while keeping the concentration under cytotoxic activity (20 µM and higher concentration [39,40,41,42,43]). Therefore, we selected 10 µM for most experiments.

### 2.4. OCR Measurements by EPR Respirometry

Oxygen measurements in vitro using EPR respirometry were based on the use of an oxygen-sensing probe, ^15^N-PDT (4-oxo-2,2,6,6-tetramethylpiperidine-d_16_-^15^N-1-oxyl), to measure variations of oxygen levels in samples, and subsequently cells’ OCR in a sealed capillary [28,43,44]. OCR was measured using a Bruker EMX-Plus spectrometer operating in X-band (9.85 GHz) and equipped with a PremiumX ultra low noise microwave bridge and a SHQ high sensitivity resonator. The EPR cavity was heated at 310 K with continuous nitrogen flow during all experiments. The respiration mixture was composed of 60 µL of previously harvested cells (stock solution 5.10^6^ cells/mL of culture medium), 40 µL of Dextran solution (20%) and 4 µL of ^15^N-PDT (2 mM), transferred into a hematocrit capillary sealed with gum. The final device was inserted into a quartz tube and put into the EPR cavity. Experimental parameters for the acquisition set in the Bruker Xenon Spin fit program were: microwave power, 2.518 mW; modulation frequency, 100 kHz; modulation amplitude, 0.005 mT; center field, 335 mT; sweep width, 1.5 mT; sweep time, 15 s. An automated “2D-field-Delay” measurement was launched 3 min after probe mixing, counting 15 points with a time delay of 60,000 ms. Data analysis was performed switching to processing mode and using “peak picking” on selected regions of the ^15^N-PDT –peaks. The final file was saved as an ASCII file to extract linewidth data at each point. ^15^N-PDT linewidth was correlated with the % of oxygen with a calibration curve. OCR corresponded to the slope of oxygen level during time. 

### 2.5. Mitochondrial Superoxide Assessment by EPR Spectrometry

Mitochondrial superoxide measurements by EPR were based on the use of Mito-TEMPO-H, a cyclic hydroxylamine able to detect superoxide in complex biological media with high sensitivity [44,45,46,47]. Thanks to its tryphenylphosphonium moiety (TPP^+^), this probe accumulates within mitochondria, enabling specific measurement of mitochondrial superoxide. Superoxide production was monitored using a Bruker EMX-Plus spectrometer operating in X-band (9.85 GHz) and equipped with a PremiumX ultra low noise microwave bridge and an SHQ high sensitivity resonator. The EPR cavity was heated at 310 K with continuous air flow during all experiments. The mixture combined 37 µL of cell suspension (stock solution: 20.10^6^ cells/mL culture medium), 0.5 µL of DTPA (100 mM), 5 µL of PBS ((1×) − pH 7.4) and 7.5 µL of Mito-TEMPO-H (1 mM). Superoxide levels were assessed by making another measurement using the same conditions but adding 2.5 µL of PEG-SOD2 (4000 U/mL) to replace 2.5 µL of PBS. PEG-SOD2 was incubated 15 min before adding the Mito-TEMPO-H probe. Previous studies have shown that the pre-incubation of cells in the presence of PEG-SOD allows the cellular uptake of the enzyme and intracellular scavenging of superoxide [48]. Mito-TEMPO-H stock solution was flushed with Argon prior and during pipetting to avoid probe oxidation. The final solution was transferred into a PTFA tube (inside diameter 0.025 in., wall thickness 0.002 in.) cutting of 12 cm using a needle and folded in 6 before being inserted into an open quartz tube. The experimental parameters for acquisition set in the Bruker Xenon Spin fit program were: microwave power, 20 mW; modulation frequency, 100 kHz; modulation amplitude, 0.1 mT; center field, 336.5 mT; sweep width, 1.5 mT; sweep time, 30.48 s. Measurements were started 3 min after probe mixing with the cells, and repeated each 40 s. An automated “2D-field-Delay” measurement was launched 3 min after probe mixing, counting 11 points with a time delay of 40,000 ms. Data analysis was performed switching to processing mode and using “Integration & derivative” and “double integration” on selected regions of the peaks. The final file was saved as an ASCII file to extract double integration (DI) data at each timepoint. Point 1 DI was subtracted from point 11 DI for each condition. Superoxide contribution was measured by subtracting mean PEGSOD2 DI to mean control DI. 

### 2.6. Tumor Models In Vivo and Treatments

All experiments involving animals were performed in accordance with the Belgian law concerning the protection and welfare of the animals and were approved by the UCLouvain ethics committee (Agreement reference: 2018/UCL/MD/021).

Six- to eight-week-old male NMRI nude mice (Charles Rivers Laboratories, Beerse, Belgium) were housed under standardized conditions of light and temperature (12-h daylight cycle, 22 ± 2 °C) before and during the experiments, and all had ad libitum access to chow pellets and water. After 1 week of acclimatization, mice were each inoculated intramuscularly (IM) in the right leg with 5 × 10^6^ PC-3 cells (100 µL cell suspension in HBSS + 100 µL Matrigel (Corning^TM^, Glendale, AZ, USA)). Tumor size was monitored three times per week using an electronic caliper, and two distances were measured, X and Y (X < Y). Tumor shape was assumed to be ellipsoidal; hence, the volume was considered π/6 × X^2^ × Y^2^. Experiments were performed 3–4 weeks following tumor inoculation when xenograft exceeded a tumor volume of >350 mm^3^. Mice were then randomly allocated to groups to have the same starting mean tumor volume before treatments.

### 2.7. In Vivo EPR Oximetry

Animals were anesthetized by inhalation of isoflurane mixed with air (21% oxygen) in a continuous flow (2 L/h), delivered by a nose cone. Induction of anesthesia was performed using 3% isoflurane. It was then stabilized at 1.5% for a minimum of 15 min before any measurement. It was previously demonstrated that this anesthesia regimen does not disturb hemodynamics in rodents [49]. A warming blanket was used for animal-body temperature regulation at 37 °C. To assess tumor oxygenation in vivo, we used EPR oximetry that allows repeated and quantitative measurements of tissue oxygenation at the same site over long periods of time [50,51,52,53]. We used charcoal (CX 0670-1; EM Sciences, Gibbstown, NJ, USA) as the oxygen sensor [54] to dynamically evaluate changes in tumor oxygenation during statin treatment. EPR spectra were recorded using an EPR spectrometer (electromagnet from Magnettech, Berlin, Germany; electronic console from Clin-EPR, Lyme, NH, USA) with a low frequency microwave bridge operating at 1.1 GHz and an extended loop resonator (Clin-EPR). A suspension of charcoal was injected into the center of the tumor 1 day before measurement (100 mg/mL; 50 µL injected, particle size of 1–25 µm). Localized EPR measurements correspond to an average of the pO_2_ values in a volume of ~10 mm^3^ [50]. For experiments, baseline values were recorded after mice were anesthetized to determine the oxygen status of tumors before the first IP injection of the treatment. Then, the effect of simvastatin and fluvastatin (20 mg/kg) was measured by following tumor 5 min after statin IP injection pO_2_ daily. Regarding the selection of the dose, statins have been used with many different dosages in mice (from 1 mg/kg/day to 100 mg/kg/day). We have started the experiment with 20 mg/kg/day in oximetry studies, in a range of doses reported in studies looking for potentiation of treatments by statins. As pO_2_ was modified, we repeated the experiments on several animals. Four mice were allocated in each group (treatment or control).

### 2.8. Irradiation Dose Assessment 

As the PC3 model was used for the first time in our laboratory, a pre-screening of irradiation doses was performed to establish a dose inducing a significant tumor growth delay without curative effect [55,56]. For this matter, male NMRI PC-3-bearing mice were randomly allocated to 5 groups with a same mean tumor volume (>350 mm^3^), and irradiated once at doses of 0–3–6–9–12 Gy with a Cesium-137 irradiator. After irradiation, tumor volume was measured 3 times a week using a caliper to assess tumor growth-delay. 

### 2.9. Tumor Growth-Delay 

The 36 Male NMRI PC-3 tumor bearing mice randomly allocated to 4 groups with the same mean tumor volume (>350 mm^3^), and irradiated once at a dose of 6 Gy at the time of maximal tumor reoxygenation (48 h after a 20 mg/kg IP simvastatin treatment) with a Cesium-137 irradiator. Groups of mice were set as follows: control group (without irradiation), simvastatin alone (without irradiation), RX (irradiation 6 Gy) and Simvastatin + RX. After irradiation, tumor volume was measured 3 times a week using a caliper to assess tumor growth-delay. For irradiation studies, 9 mice were used in each group.

### 2.10. Statistics

Data are represented as means ± SEM. All experiments were performed in triplicate or more. Student’s *t*-test was applied for OCR, EPR and mitochondrial superoxide measurements’ experiments, whereas one-way ANOVA or two-way ANOVA were performed other experiments.

## 3. Results

### 3.1. OCR in Prostate Cancer Cells Is Decreased by Simvastatin and Fluvastatin, But Is Unchanged after Pravastatin, Rosuvastatin or Atorvastatin Exposure

**Figure 2:**
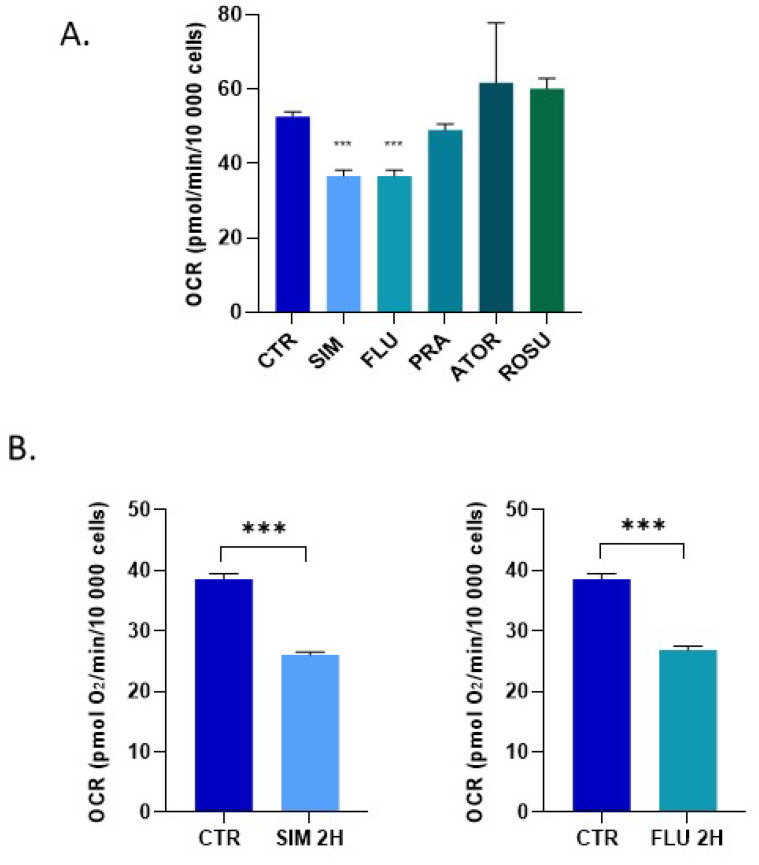
Impact of statins on the oxygen consumption rate (OCR) by prostate cancer cells as measured by Seahorse XF96. (**A**) effect of a 24 h exposure to five different statins (10 μM) on the OCR of PC-3 cells; (**B**) effect of a 2 h exposure to simvastatin and fluvastatin (10 μM) on the OCR of PC-3 cells. Bars represent means ± SEM (pmol O_2_/min/10,000 cells), (***) *p* < 0.001, one-way ANOVA & Student’s *t*-test, *n* = 5.

A first screening using the Seahorse XF96 technology was performed on PC-3 cells that revealed that a 24 h exposure of prostate cancer cells to a concentration of 10 µM simvastatin and fluvastatin significantly decreased the basal respiration (<0.005, Figure 2A). In contrast, pravastatin, rosuvastatin and atorvastatin did not significantly alter the OCR (Figure 2A). Fluvastatin and simvastatin were further considered for additional experiments. We found that a significant reduction was also observed using Seahorse XF96 after a 2 h exposure (Figure 2B). We also found that a lower concentration (2.5 µM) significantly decreased basal OCR by 24% and 25.8% for simvastatin and fluvastatin, respectively. 

**Figure 3:**
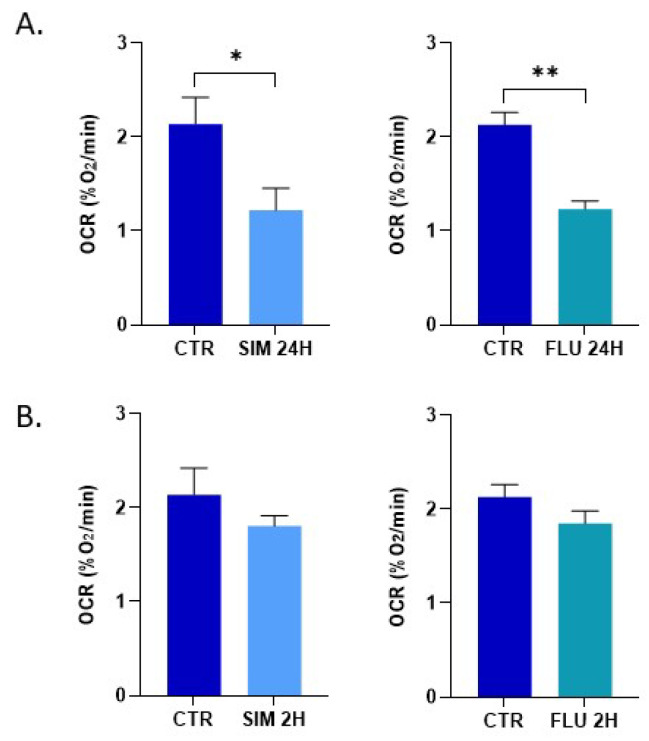
Impact of statins on the oxygen consumption rate (OCR) by prostate cancer cells as measured by EPR respirometry. (**A**) effect of a 24 h exposure to simvastatin and fluvastatin (10 μM) on the OCR of PC-3 cells; (**B**) effect of a 2 h exposure to simvastatin and fluvastatin (10 μM) on the OCR of PC-3 cells. Bars represent means ± SEM (% O_2_/min), (*) *p* < 0.05, (**) *p* < 0.01, Student’s *t*-test, *n* = 3.

In addition, we used in vitro EPR respirometry to assess the OCR of cancer cells. While the results obtained after a 24-h exposure were consistent between Seahorse XF96 and EPR assessments, we did not observe a significant effect of either statin after a 2 h exposure time using EPR respirometry (Figure 3).

### 3.2. Modulation of Mitochondrial Superoxide Production by Simvastatin and Fluvastatin

**Figure 4:**
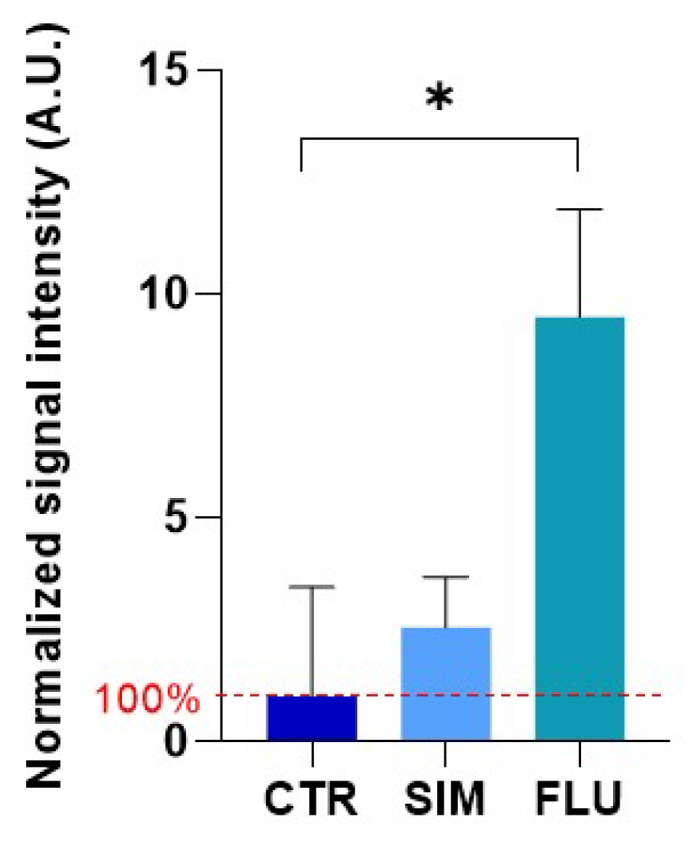
Impact of statins on the mitochondrial superoxide level of PC-3 cells exposed 24 h to simvastatin and fluvastatin (10 µM). Mitochondrial superoxide was measured using mitoTEMPO-H as an EPR sensor. Superoxide contribution to the signal was measured by making the difference between the signal intensities recorded in the absence and in the presence of PEGSOD2. Bars represent means ± SEM (A.U), (*) *p* < 0.05, one-way ANOVA, *n* = 3.

Because a dysfunction of the mitochondrial electron transport chain may lead to an increase in superoxide production [57,58], we evaluated the potential change of mitochondrial superoxide levels in cells exposed for 24 h to 10 µM simvastatin or fluvastatin. The level of superoxide was significantly increased after the exposure of prostate cancer cells to fluvastatin (Figure 4). While there was a trend for an increase in mitochondrial superoxide levels after simvastatin exposure, it did not reach statistical significance (Figure 4).

### 3.3. Simvastatin and Fluvastatin Alleviate Tumor Hypoxia in Prostate Cancer Models

**Figure 5:**
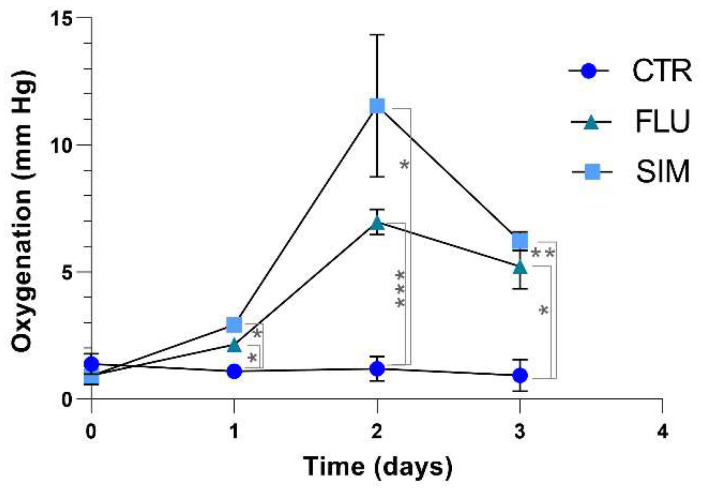
Effect of daily treatment with statins (20 mg/kg) on tumor oxygenation as measured by EPR oximetry. Simvastatin (44 µmol/kg), fluvastatin (46 µmol/kg). Data are shown as means ± SEM, (*) *p* < 0.05, (**) *p* < 0.01, (***) *p* < 0.001, two-way ANOVA, *n* = 4.

Because prostate cancer cells are sparing oxygen when exposed to simvastatin and fluvastatin in vitro, we anticipated that statin-based treatments could lead to a reoxygenation of tumors in vivo. The PC3 prostate tumor model was found to be highly hypoxic, as the initial pO_2_ before treatment was lower than 2 mm Hg (Figure 5). Daily treatment with simvastatin and fluvastatin led to a sharp increase in tumor oxygenation 48 h and 72 h after having treatment initiation (Figure 5). The slight decrease observed at day 3 is likely due to the decrease in perfusion due to the persistent growth of the tumor.

### 3.4. Does the Combination Statin/Irradiation Lead to an Improved Response to Radiotherapy?

**Figure 6:**
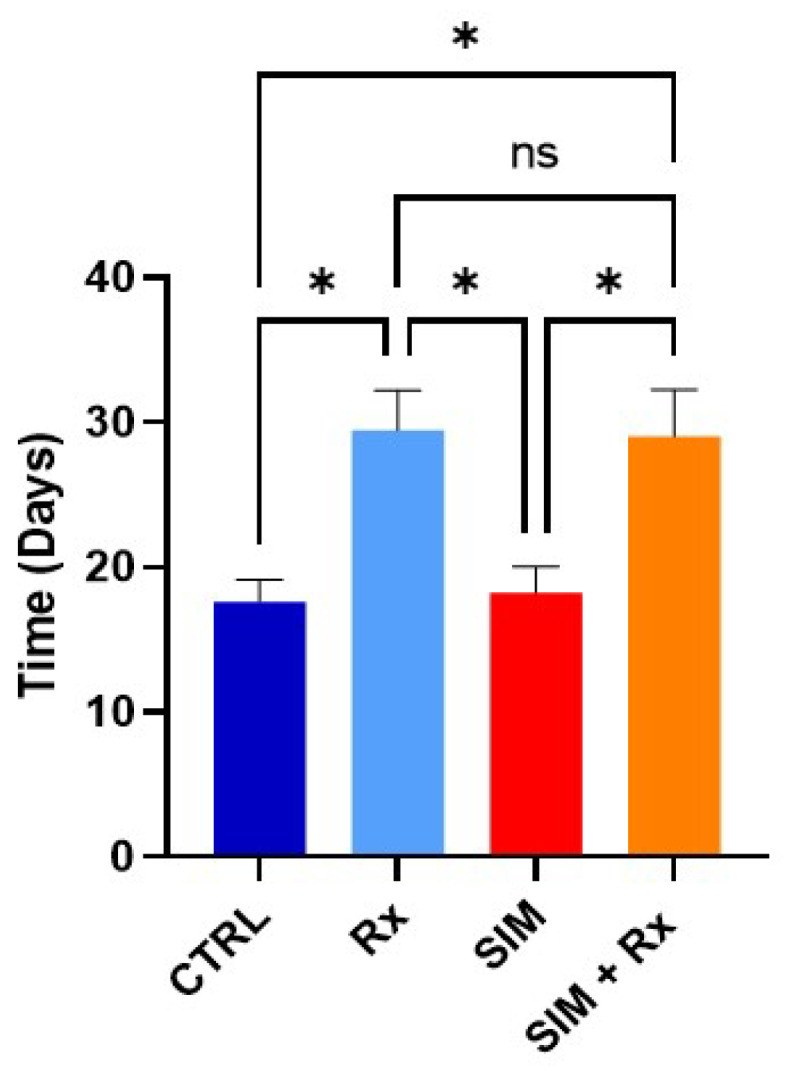
Effect of daily treatment with Simvastatin (20 mg/kg) delivered at the time of maximal tumor reoxygenation on PC-3 tumor growth. Data are shown as means ± SEM of tumor growth to achieve a 1200 mm^3^ tumor volume. (*) *p* < 0.05, two-way ANOVA *n* = 9/group.

For this experiment, simvastin was selected because the level of oxygen in tumors reached after treatment was higher than the one observed after fluvastatin. Despite promising in vitro and in vivo tumor reoxygenation results, Simvastatin treatment at the time of maximal reoxygenation did not improve PC-3 tumor growth delay in mice after irradiation compared to the “RX alone” group (Figure 6).

## 4. Discussion

The negative effect of tumor hypoxia on the response to photon-based radiation therapy has been demonstrated in a vast body of experimental works and clinical studies as reviewed in [20,21,22,23,24]. Several strategies have been designed to overcome this source of radioresistance, including decreasing tumor hypoxia, chemical radiosensitization of hypoxic cells, or preferential killing of the resistant cell population by using a redistribution of the radiation dose. The most straightforward approach to decrease hypoxia is to combine a radiation protocol with the administration of an approved drug acting either on oxygen consumption and/or on oxygen delivery. Illustrative successful examples in tumor models include the use of steroidal and non-steroidal anti-inflammatory agents [29] and metformin [31]. In this respect, if statins could be found to be potential radiosensitizers, they would be particularly attractive regarding their massive use in the population to prevent cardiovascular diseases. 

Because a meta-analysis described a beneficial effect of statins on prostate cancer patients treated with RT [18], it seemed crucial to investigate the mechanisms underlying the radiosensitizing properties of statins. Intriguingly, a recent study suggested that simvastatin (µM range) combined with irradiation slightly affects cell growth, clonogenic survival, and the DNA repair capacity of the breast cancer cell line MCF-7 in 2D-culture, but, surprisingly, not in 3D-culture [59]. Another study described that simvastatin (10 to 500 µM) compromises DNA double-strand break repair by triggering the expression of histone 2A family member X (γ-H2AX) and phospho-checkpoint kinase 1 (p-CHK1), suggesting an underlying mechanism for this radiosensitization of prostate cancer cells (PC3 model) [60]. It was also found that simvastatin (1 to 5 µM) enhanced the in vitro radiation sensitivity of different colorectal cancer cell lines [61]. In the present study, we explored another potential mechanism of radiosensitization, namely a potential change in tumor cell metabolism that should impact the tumor microenvironment. Our initial hypothesis of a change in OCR was verified in prostate cancer cells exposed to simvastatin or fluvastatin (Figure 2). However, contrarily to our expectation, we did not observe a “class effect”, as the OCR was not significantly changed when exposing the prostate cancer cells to pravastatin, rosuvastatin or atorvastatin (Figure 2). Moreover, as rosuvastatin and atorvastatin are more potent inhibitors of HMG-CoA reductase than simvastatin [62], this suggests that the effect observed on prostate cancer cells is not primarily mediated by a reduction of the synthesis of Coenzyme Q10 (Complex III of the mitochondrial electron transport chain). In the research on mechanisms associated with muscle toxicity, it has been described that statins inhibit the activity of enzyme Complexes I and III of the electron transport chain, an inhibition that is dependent on individual statin [63]. Identifying the exact mechanism responsible for the change in the OCR of cancer cells will require further investigation. It is worth noting that we observed consistent results in OCR decrease using Seahorse XF96 and EPR respirometry after a 24 h exposure, but not after 2 h of exposure (Figure 2 and Figure 3). While Seahorse XF96 is applied on adherent cells, EPR respirometry requires cell detachment. A previous study revealed that cell detachment with trypsin may lead to slight change in cell respiration [64]. Interestingly, the consequence of the induced mitochondrial dysfunction in terms of superoxide production was different between statins. While simvastatin and fluvastatin had a comparable impact on OCR (Figure 2 and Figure 3), the level of mitochondrial superoxide produced was higher after fluvastatin treatment than after simvastatin exposure (Figure 4).

An expected consequence of the change in the OCR of prostate cancer cells was the alleviation of tumor hypoxia. To evaluate the impact of statin treatment on the PC-3 cancer model in vivo, we selected EPR oximetry because this technique has the unique capabilities to acquire repeated measurements at the same site over long periods of time and to detect subtle variations of tissue oxygenation [51,53]. Consistently with our observations done in vitro, the increase in tumor oxygenation did not occur acutely after the administration of the statins. Maximal reoxygenation was observed 48 h after the first injection (Figure 5). It is worth noting that the pO_2_ values increased from about 2 mmHg before treatment to 7 mmHg or 12 mmHg, after 2 days of treatment with fluvastatin and simvastatin, respectively. It is well established that the Oxygen Enhancement Ratio (i.e., the ratio of radiation dose to observe a same biological effect) dramatically varies between 1 and 10 mm Hg [23,65,66]. We therefore anticipated that this increase in tumor oxygenation would be beneficial in terms of radiosensitization if the irradiation would have been applied during the time window identified by EPR oximetry. This in vivo irradiation protocol to assess the effectiveness of statins on the response to irradiation was performed after identifying the optimal dose of irradiation with a significant tumor growth delay without a curative effect.

Despite statins’ promising increase in PC-3 tumor reoxygenation, simvastatin combined with irradiation did not induce any tumor growth-delay compared to the RT alone group (Figure 6). In previous studies, an increase in oxygenation due to a decrease in OCR resulted in an increase in radiation response, as reviewed in [33]. The elucidation of the reasons for the absence of radiosensitization observed in this tumor model will require further investigation.

A limitation of our study relies on the statin dosage that has been used in vitro to study the effect of oxygen utilization. While our present study was performed using concentrations consistent with those used in other studies evaluating the impact of statins on tumor cell response [3,4,5,6,59,60,61], their relevance for immediate translation in humans is debatable. A publication performed a systematic literature search identifying in vitro experiments, where mechanistic insights behind the pleiotropic effects of statins were claimed [67]. In this paper, the authors concluded that the pleiotropic effects of statins were only detected at statin concentrations of 1–50 µmol/L. This contrasts with the maximal concentration (Cmax) of statin found in the human serum (30 nmol/L for simvastatin after a dose of 40 mg, and 40 nmol/L for atorvastatin after a dose of 20 mg) [67]. Here, taking the example of simvastatin, we used an in vitro concentration of 10 µM in most experiments, and we also observed an effect on OCR at a concentration of 2.5 µM. This represents about eighty times the Cmax observed in humans. Regarding the dose used in vivo, we administered a dose of 20 mg/kg of simvastatin, while the maximal dose in humans is 80 mg per day for an adult, representing 1 mg/kg for an adult of 80 kg body weight. This represents approximately 20 times the dose used in humans. In other words, the effects observed on cancer cell metabolism should be considered as a proof-of-concept, and any translation for human studies should be taken with caution considering the difference in statins concentrations achieved after standard dosage. Another limitation of our study is that we did not use an orthotopic model of prostate cancer. While the hypoxic environment was recapitulated as demonstrated by the quantitative measurement of pO_2_, we cannot exclude that the results would have been slightly different using an orthotopic implantation of the prostate cancer cells.

## Data Availability

All results are reported in the manuscript.
